# Omicron Booster in Ancestral Strain Vaccinated Mice Augments Protective Immunities Against Both Delta and Omicron Variants

**DOI:** 10.3389/fimmu.2022.897879

**Published:** 2022-07-06

**Authors:** Liqiu Jia, Yang Zhou, Shaoshuai Li, Yifan Zhang, Dongmei Yan, Wanhai Wang, Wenhong Zhang, Yanmin Wan, Chao Qiu

**Affiliations:** ^1^ Department of Infectious Diseases, Shanghai Key Laboratory of Infectious Diseases and Biosafety Emergency Response, National Medical Center for Infectious Diseases, Huashan Hospital, Shanghai Medical College, Fudan University, Shanghai, China; ^2^ Department of Laboratory Medicine, Shanghai Public Health Clinical Center Fudan University, Shanghai, China; ^3^ Department of Immunology, School of Basic Medical, Jiamusi University, Jiamusi, China; ^4^ Clinical Laboratory, The First Affiliated Hospital of Zhengzhou University, Zhengzhou, Hehan, China; ^5^ National Clinical Research Center for Aging and Medicine, Huashan Hospital, Fudan University, Shanghai, China; ^6^ Key Laboratory of Medical Molecular Virology (MOE/MOH), Shanghai Medical College, Fudan University, Shanghai, China; ^7^ Department of Radiology, Shanghai Public Health Clinical Center, Fudan University, Shanghai, China; ^8^ Institutes of Biomedical Sciences & Shanghai Key Laboratory of Medical Epigenetics, Fudan University, Shanghai, China

**Keywords:** Omicron-matched vaccine, booster vaccination, cross-reactivity, T cell, antibody

## Abstract

A booster vaccination is called for constraining the evolving epidemic of SARS-CoV-2. However, the necessity of a new COVID-19 vaccine is currently unclear. To compare the effect of an Omicron-matched S DNA vaccine and an ancestral S DNA vaccine in boosting cross-reactive immunities, we firstly immunized mice with two-dose of a DNA vaccine encoding the spike protein of the ancestral Wuhan strain. Then the mice were boosted with DNA vaccines encoding spike proteins of either the Wuhan strain or the Omicron variant. Specific antibody and T cell responses were measured at 4 weeks post boost. Our data showed that the Omicron-matched vaccine efficiently boosted RBD binding antibody and neutralizing antibody responses against both the Delta and the Omicron variants. Of note, antibody responses against the Omicron variant elicited by the Omicron-matched vaccine were much stronger than those induced by the ancestral S DNA vaccine. Meanwhile, CD8^+^ T cell responses against both the ancestral Wuhan strain and the Omicron strain also tended to be higher in mice boosted by the Omicron-matched vaccine than those in mice boosted with the ancestral S DNA vaccine, albeit no significant difference was observed. Our findings suggest that an Omicron-matched vaccine is preferred for boosting cross-protective immunities.

## Introduction

The highly mutated SARS-CoV-2 Omicron (B.1.1.529) variant has been shown to substantially evade the neutralizing antibody responses elicited by current vaccines and early pandemic Alpha, Beta, Gamma, or Delta variant (1–9). A third dose of either homologous or heterologous COVID-19 vaccine was reported to enhance neutralizing antibody responses against the Omicron variant (3, 4, 10–15). However, the magnitudes of neutralizing activities towards Omicron after the booster dose were still far lower compared to earlier variants of concern (3, 5, 11, 16–19). Therefore, more effective vaccines or vaccination strategies are urgent in need to control the evolving pandemic of SARS-CoV-2 (20–22). Recently preprinted studies demonstrated that Omicron infection of previously vaccinated individuals could boost broadly neutralizing antibodies against different SARS-CoV-2 variants (23, 24), suggesting that the spike protein of the Omicron variant might serve as a good candidate antigen for a new COVID vaccine. Meanwhile, contradictory findings suggest that Omicron-matched vaccination shows no superiority in protection compared to immunization with current vaccines (25–28), which exaggerates concern about the effect of original antigenic sin caused by exposures to ancestral SARS-CoV-2 variants (29).

To investigate the effect of using Omicron-matched spike protein as a booster antigen, we firstly immunized mice with two-dose of a DNA vaccine encoding the spike protein of the ancestral Wuhan strain. Then the mice were boosted with DNA vaccines encoding spike proteins of either the Wuhan strain or the Omicron variant. Our data showed that the Omicron S DNA vaccine boosted cross-reactive antibody and T cell responses more efficiently than the ancestral S DNA booster vaccine.

## Materials and Methods

### Ethics Statement

All experiments and methods were performed in accordance with relevant guidelines and regulations. Mice experiments were reviewed and approved by the Research Ethics Review Committee of Shanghai Public Health Clinical Center.

### Constructions and Preparation of Candidate DNA Vaccine Encoding the Full-Length Spike Proteins of Wuhan or Omicron Strain

The full-length *s* genes of the SARS-CoV-2 Wuhan and Omicron strain were optimized according to the preference of human codon usage and synthesized by Genewiz (Genewiz Biotech Co., Ltd., Suchow, China). The codon optimized spike genes were subcloned into the pJW4303 eukaryotic expression vector (Kindly gifted by Dr. shan Lu’s laboratory at the University of Massachusetts). The sequence of insertion was confirmed by Sanger sequencing (Sangon Biotech Co., Ltd., Shanghai, China). The recombinant plasmids for mouse vaccination were prepared using an EndoFree Plasmid Purification Kit (Cat# 12391, Qiagen, Hilden, USA).

### Mouse Vaccination

**Figure 1:**
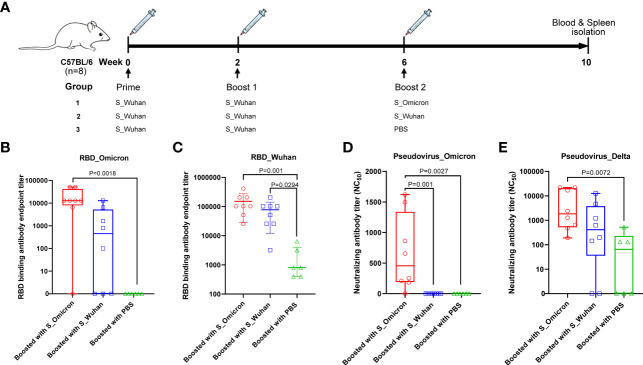
Boosting with the Omicron_S DNA vaccine elicited cross-protective antibodies in mice. **(A)** Schematic illustration of the vaccination schedule. Female C57BL/6J mice were injected intramuscularly with 20μg of the S_Wuhan DNA vaccine at Week 0 and Week 2. Subsequently, the mice were boosted with 50μg of either the S_Omicron (n=8) or the S_Wuhan (n=8) DNA vaccines at Week 6. The control group was boosted with PBS (n=6). Four weeks after the final vaccination, the mice were euthanized. Peripheral blood and splenocytes were collected on spot for assays of S protein specific immune responses. **(B)** Titers of IgG binding to the RBD protein of the Omicron variant. Data is shown as median with IQR (Interquartile range). **(C)**Titers of IgG binding to the RBD protein of the Wuhan strain. Data is shown as mean ± SD. **(D)** Titers of neutralizing antibodies against the pseudo-virus of the Omicron variant. Data is shown as median with IQR. **(E)** Titers of neutralizing antibodies against the pseudo-virus of the Delta variant. Data is shown as median with IQR. Antibody titers were measured by ELISA assays with duplicated wells for each dilution of each sample and calculated as the reciprocal of endpoint titer. The neutralizing antibody assay was repeated twice. Comparisons among three groups were conducted using the method of one-way ANOVA.

Female C57BL/6J mice, 6-8-week-old, were purchased from Vital River Laboratory Animal Technology Co., Ltd. (Beijing, China) and housed in the SPF animal facility of Shanghai Public Health Clinical Center. The schedule of vaccination is shown in **Figure 1A**. Briefly, 20μg of the S_Wuhan DNA vaccine was injected intramuscularly into each mouse at Week 0 and Week 2. Subsequently, the mice were boosted with 50μg of either the S_Omicron or the S_Wuhan DNA vaccine at Week 6. The regimen is designed based on our previous findings, which show that increasing DNA dosage for the 3^rd^ vaccination can ensure the efficiency of *in vivo* antigen expression (30). The control group was boosted with PBS. Four weeks after the final vaccination, the mice were euthanized. Peripheral blood and splenocytes were collected for assays of S protein specific immune responses.

### Detection of RBD Specific Binding Antibodies

An in-house enzyme-linked immunosorbent assays (ELISA) were developed to measure RBD of Wuhan or Omicron strain of SARS-CoV-2 specific binding antibodies. High-binding 96-well EIA plates (Cat# 9018, Corning, USA) were coated with purified SARS-CoV-2 RBD protein of Wuhan strain (Cat# 40592-V08B, Sino Biological, China) or RBD protein of Omicron strain (Cat# 40592-V08H122, Sino Biological, China) at a final concentration of 1µg/ml in carbonate/bi-carbonate coating buffer (30mM NaHCO_3_,10mM Na_2_CO_3_, pH 9.6). After coating overnight at 4°C, the plates were blocked with 1×PBS containing 5% milk for 1 hour at 37°C. Subsequently, 100μl of serial dilutions of mouse serum was added to each well. After 1 h incubation at 37°C, the plates were washed with 1×PBS containing 0.05% Tween 20 for 5 times. Then, 100μl of an HRP labeled goat anti-mouse IgG antibody (Cat# 115-035-003, Jackson Immuno Research, USA) diluted in 1×PBS containing 5% milk were added to each well and incubated for 1 hour at 37°C. After a second round of wash, 100μl of TMB substrate reagent (Cat# MG882, MESGEN, China) was added to each well. 6 minutes later, the color development was stopped by adding 100μl of 1M H_2_SO_4_ to each well and the values of optical density at OD450nm and OD630nm were measured using 800 TS microplate reader (Cat# 800TS, Biotek, USA).

### Flow Cytometry Assay

S specific T cell responses in splenocytes were detected by flowcytometry assay. Briefly, fresh splenocytes were prepared and stimulated with R10 containing synthesized peptides covering the full-length spike protein of Wuhan strain (152 peptides in total, 0.66 μg/ml per peptide) or peptides covering the fragments of spike protein containing mutations specific to Omicron variant (36 peptides in total, 0.66 μg/ml per peptide) in round-bottom 96-well plates. The detailed information about the synthesized peptides is shown in **Supplementary Table 1**. 2 hours later, brefeldin A were added to each well at final concentrations of 1 μg/ml. After 12 hours, the cells were collected and stained sequentially with Live/Dead dye (Fixable Viability Stain 510, Cat# 564406, BD Pharmingen) for 15 min at room temperature, surface markers (PE/Cyanine7-labeled anti-mouse CD3, Cat# 100220, BioLegend; APC-labeled anti-mouse CD4, Cat# 100412, BioLegend; PE-labeled anti-mouse CD8, Cat# 100708, BioLegend) for 30min at 4 ˚C and intracellular markers (BV421-labeled anti-mouse IFN-γ, Cat# 505830, BioLegend; FITC-labeled anti-mouse IL-2, Cat# 503806, BioLegend; BV711-labeled anti-mouse TNF-α, Cat# 506349, BioLegend) for 30min at 4 °C. After washing, the stained cells were resuspended in 200 μl 1×PBS and analyzed using a BD LSRFortessa™ Flow Cytometer. The data were analyzed using the FlowJo 10 software (BD Biosciences, USA). The gating strategy was shown in **Supplementary Figure 1**. The percentages for specific T-cell populations were defined as the percentages of cytokines producing CD4^+^ or CD8^+^ cells in all CD4^+^ or CD8^+^ T cells.

### SARS-CoV-2 Pseudovirus Neutralization Assay

VSV-backboned SARS-CoV-2 pseudo-viruses were prepared according to a reported method (31). The neutralization assay was conducted by following the previously described procedure (31, 32). Briefly, 100μl of serially diluted mice sera were added into 96-well cell culture plates. Then, 50μl of pseudo-viruses with a titer of 13000 TCID_50_/ml were added into each well and the plates were incubated at 37°C for 1 hour. Next, Vero cells were added into each well (2×10^4^ cells/well) and the plates were incubated at 37°C in a humidified incubator with 5% CO_2_. 24 hours later, luminescence detection reagent (Bright-Glo™ Luciferase Assay System, Promega, USA) was added to each well following the manufacturer`s instruction. The luminescence was measured using a luminescence microplate reader (GloMax^®^ Navigator Microplate Luminometer, Promega, USA) within 5 minutes. The Reed-Muench method was used to calculate the virus neutralization titer. Antibody neutralization titers were presented as 50% maximal inhibitory concentration (IC_50_).

### Statistical Analysis

All statistical analyses were performed using GraphPad Prism 9 (GraphPad Software, Inc., La Jolla, CA, USA). Comparisons among three groups were conducted by the method of one-way ANOVA. P<0.05 was considered as statistically significant.

## Results

### Boosting With a DNA Vaccine Encoding the Spike Protein of Omicron Variant Elicited Cross-Reactive Antibody Responses in Mice

As afore mentioned, female C57BL/6J mice were immunized with 20μg of the S_Wuhan DNA vaccine at Week 0 and Week 2. Subsequently, the mice were boosted with 50μg of DNA vaccines expressing the S proteins of either the Omicron variant or the Wuhan strain (**Figure 1A**) at Week 6. Sera were collected at the 4^th^ week post the last vaccination to measure RBD specific binding antibodies by a method of ELISA (**Figures 1B, C**). Our data showed that booster vaccination with S_Wuhan DNA vaccine significantly enhanced the binding antibody response to RBD protein of ancestral Wuhan strain compared with that of PBS group. A booster shot of the S_Omicron DNA vaccine significantly increased RBD binding antibody responses against both the ancestral Wuhan and the Omicron variant compared to that of the PBS control. Additionally, the mean titer of RBD binding antibodies against the Wuhan and the Omicron variant tended to be higher in the group boosted with the S_Omicron DNA vaccine than the group boosted with the S_Wuhan DNA vaccine, although the difference is not statistically significant.

Then the neutralizing antibody titers were assessed using a VSV-backboned SARS-CoV-2 pseudo-virus assay. Similar to the results of binding antibody assays, we found that a booster shot of the S_Omicron DNA vaccine elicited significantly stronger neutralizing antibody responses against the Omicron variant compared with booster doses of the S_Wuhan DNA vaccine and the PBS control (**Figure 1D**). To further investigate the impact of S_Omicron DNA vaccine against SARS-CoV-2 variants, neutralizing antibodies were measured for against Delta variant, which was an important variant of concern (**Figure 1E**). The results showed that a booster with the S_Omicron DNA vaccine significantly enhanced the neutralizing antibody responses against the Delta variant compared with the PBS control. In addition, the mean titer of neutralizing antibodies against the Delta variant also tended to be higher in the group boosted with the S_Omicron DNA vaccine than the group boosted with the S_Wuhan DNA vaccine, although the difference is not statistically significant.

### The S_Omicron DNA Vaccine Booster Improved T Cell Responses Against Both the Wuhan Strain and the Omicron Variant

**Figure 2:**
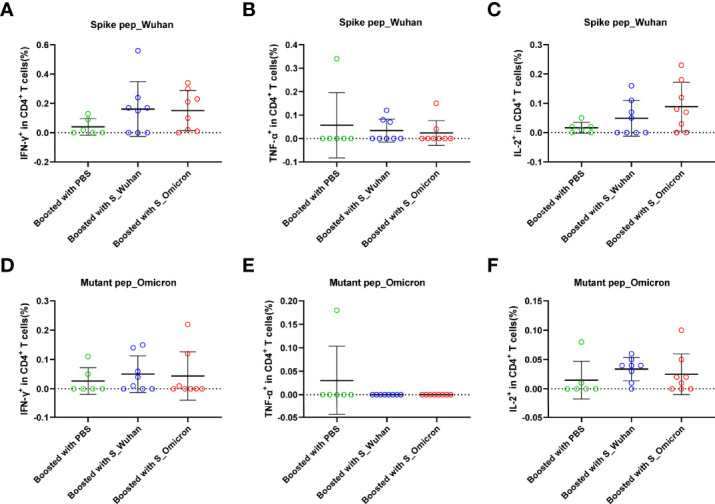
Boosting with the S_Omicron DNA vaccine showed no improvement of mono-functional CD4^+^ T cell responses. Comparisons of IFN-γ **(A)** TNF-α **(B)** and IL-2 **(C)** producing CD4^+^ T cell response to the spike protein of ancestral Wuhan strain. Comparisons of IFN-γ **(D)** TNF-α **(E)** and IL-2 **(F)** producing CD4^+^ T cell response to the spike protein of Omicron variant. Data are shown as mean ± SD. Statistical analyses were performed using the method of one-way ANOVA. S_Omicron DNA vaccine, n=8; S_Wuhan DNA vaccine, n=8; PBS control, n=6.

**Figure 3:**
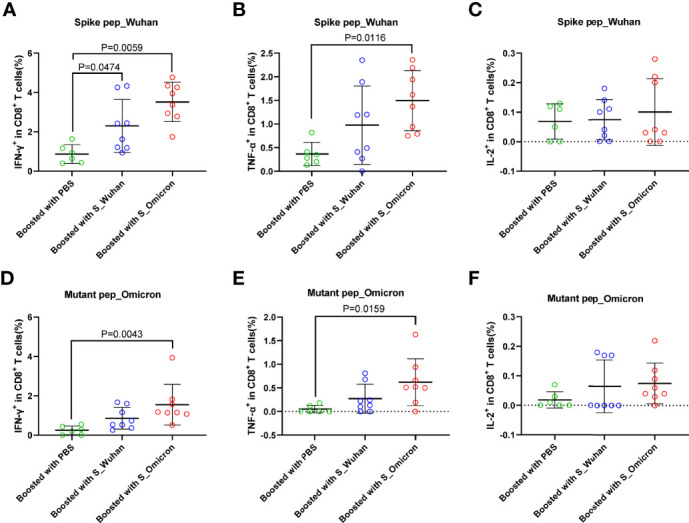
Boosting with the S_Omicron DNA vaccine enhanced mono-functional CD8^+^ T cell responses. Comparisons of IFN-γ **(A)** TNF-α **(B)** and IL-2 **(C)** producing CD8^+^ T cell response to the spike protein of ancestral Wuhan strain. Comparisons of IFN-γ **(D)** TNF-α **(E)** and IL-2 **(F)** producing CD8^+^ T cell response to the spike protein of Omicron variant. Data are shown as mean ± SD. Statistical analyses were performed using the method of one-way ANOVA. S_Omicron DNA vaccine, n=8; S_Wuhan DNA vaccine, n=8; PBS control, n=6.

**Figure 4:**
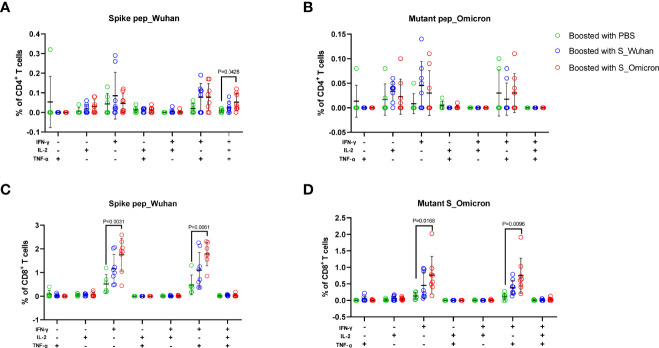
Boosting with the S_Omicron DNA vaccine improved polyfunctional T cell responses against spike protein of both the Wuhan and the Omicron variant. The Multi-functionality of spike protein specific T cells was delineated *via* measuring the secretions of IFN-γ, TNF-α and IL-2. **(A)** Comparisons of the polyfunctional CD4^+^ T cell responses against the spike protein of the ancestral Wuhan variant. **(B)** Comparisons of the polyfunctional CD4^+^ T cell responses against the spike protein of the Omicron variant. **(C)** Comparisons of the polyfunctional CD8^+^ T cell responses against the spike protein of the ancestral Wuhan strain. **(D)** Comparisons of the polyfunctional CD8^+^ T cell responses against the spike protein of the Omicron variant. Data are shown as mean ± SD. Statistical analyses were performed using the method of one-way ANOVA. S_Omicron DNA vaccine, n=8; S_Wuhan DNA vaccine, n=8; PBS control, n=6.

At the 4^th^ week post the booster vaccinations, mouse splenocytes were freshly isolated and stimulated with peptides covering the full-length spike protein of Wuhan strain or the fragments of Omicron spike protein containing mutations. Specific T cell responses were assessed by intracellular cytokine staining assays (**Figure 2** and **Figure 3**). Our data showed that a booster vaccination of the S_Wuhan and the S_Omicron DNA vaccine failed to improve IL-2^+^, TNF-α^+^ or IFN-γ^+^ CD4^+^ T cell responses against the spikes protein of both the Wuhan and the Omicron variant as compared to those of the PBS control (**Figure 2**). Multifunction analyses showed that the booster vaccination of the S_Omicron DNA vaccine significantly enhanced IL-2^+^TNF-α^+^IFN-γ^+^ polyfunctional CD4^+^ T cell responses against the spike protein of Wuhan strain (**Figure 4A**), but it failed to improve the polyfunctional CD4^+^ T cell responses against the Omicron variant (**Figure 4B**).

In comparison with CD4^+^ T cell responses, a booster with the S_Wuhan DNA vaccine significantly augmented the IFN-γ^+^ CD8^+^ T cell responses against the spike protein of Wuhan strain as compared to the PBS control (**Figure 3A**). While, a booster with the S_Omicron DNA vaccine significantly enhanced IFN-γ^+^CD8^+^ (**Figures 3A, D**) and TNF-α^+^CD8^+^ T responses (**Figures 3B, E**) against both the Wuhan and the Omicron variant. The IL-2^+^CD8^+^ T cell responses against the Wuhan and the Omicron variant were comparable among all three groups (**Figures 3C, F**). The polyfunctional (IL-2^-^TNF-α^+^IFN-γ^+^) CD8^+^ T cell responses against the spike protein of both the Wuhan and the Omicron variant were significantly improved in mice boosted with the S_Omicron DNA vaccine as compared to those boosted with PBS control (**Figures 4C, D**). However, no significant difference was observed between the polyfunctional CD8^+^ T cell responses of mice boosted with the S_Omicron and the S_Wuhan DNA vaccines.

## Discussion

Extensive mutations in the spike protein render the Omicron variant more likely to escape current vaccines than ancestral SARS-CoV-2 variants (33, 34). A third dose can reduce the risk of hospitalization due to Omicron, however, the effectiveness may diminish quickly (35). The necessity and benefit of an Omicron vaccine is now under debate. Comparisons between current vaccines and Omicron-matched vaccines can provide valuable information on development of new COVID-19 vaccines.

Given that a massive population have been previously infected by and/or vaccinated against SARS-CoV-2 globally, the selection of a proper booster vaccine is critical for eliciting optimal protective immunities against mutated variants. As a promising nucleic vaccine platform, DNA vaccines offer a simple but effective mean to induce both protective humoral and cellular immune responses. Although the immunogenicity of DNA vaccines is relatively weak in human, it has unique advantages such as the low cost and extraordinary stability (36). Hence, in this study, we compared the booster effect of a Wuhan S DNA vaccine and an Omicron S DNA vaccine in mice primed with two-dose of a Wuhan S DNA vaccine. In consistent with a previous study (25), our results show that a booster with Omicron-matched vaccine can elicit stronger inhibitory antibody responses against the Omicron variant. Meanwhile, we found that the mice boosted with the Omicron S DNA vaccine also maintained relatively higher RBD binding antibody and neutralizing antibody responses against the Delta variant. Our finding is consistence with most recent studies showing that Omicron infection alone did not induce superior cross-reactive antibody responses, while Omicron infection in previously vaccinated individuals can significantly enhance cross-protective neutralizing antibody responses (23, 37). These observations suggest that a booster of an Omicron-matched vaccine might help to boost broadly neutralizing antibody responses. Of note, in this study, we did not monitor the antibody responses for a longer term, therefore further experiments are needed to compare the durability of protective antibody responses between boosters of the Omicron matched vaccine and the ancestral variant matched vaccine.

In addition to humoral responses, cross-reactive T cell responses have also been observed in previously infected or vaccinated people (19, 38–40), which hold the potential to protect against severe COVID-19. Here we show that the Omicron-matched vaccine is more efficient in boosting cross-reactive CD8^+^ T cell responses, while the CD4^+^ T cell responses are relatively difficult to improve. This phenomenon could be explained by previous observations suggesting DNA vaccines are more effective in eliciting CD8^+^ than CD4^+^ T cell responses (41). As a recent study demonstrates that the Omicron variant might escape T cell immunities in some individuals with prior infection or vaccination (42), a booster vaccination that can improve T cell cross-reactivity will very likely help to protect against emerging SARS-CoV-2 variants.

We note the following limitations in our study. First, we were not able to conduct a live-virus challenge experiment, which was primarily due to the lack of access to a BSL-3 lab. Therefore, we do not know whether the observed improvements of humoral and cellular immunities can be translated into superior protection. Second, the results were generated using a mouse model, which might not completely mimic the characteristics of human immune responses. The Omicron variant challenge studies in animals and vaccination in humans will be required for corroboration.

## Data Availability Statement

The original contributions presented in the study are included in the article/**Supplementary Material**. Further inquiries can be directed to the corresponding authors.

## Ethics Statement

The animal study was reviewed and approved by The Research Ethics Review Committee of Shanghai Public Health Clinical Center.

## Author Contributions

CQ, YMW, and WHZ designed the study. LQJ, YZ, SSL, YFZ, and YMW conducted the experiments. YMW, LQJ, and YZ analyzed the data and drafted the manuscript. CQ, WHZ, DMY, and WHW revised the manuscript. All authors contributed to the article and approved the submitted version.

## Funding

This work was partly supported by the National Natural Science Foundation of China (Grant No. 81971559, 31872744), the Science and Technology Commission of Shanghai Municipality (Grant No. 21NL2600100, HS2021SHZX001, 20dz2260100), the major project of Study on Pathogenesis and Epidemic Prevention Technology System (Grant No. 2021YFC2302500) by the Ministry of Science and Technology of China, and Key Discipline Construction Plan from Shanghai Municipal Health Commission (GWV-10.1-XK01).

## Conflict of Interest

The authors declare that the research was conducted in the absence of any commercial or financial relationships that could be construed as a potential conflict of interest.

## Publisher’s Note

All claims expressed in this article are solely those of the authors and do not necessarily represent those of their affiliated organizations, or those of the publisher, the editors and the reviewers. Any product that may be evaluated in this article, or claim that may be made by its manufacturer, is not guaranteed or endorsed by the publisher.
