# Supplementary material for: Omicron Booster in Ancestral Strain Vaccinated Mice Augments Protective Immunities Against Both Delta and Omicron Variants
Source: Front Immunol. 2022 Jul 6;13:897879. doi: 10.3389/fimmu.2022.897879 (PMC9298979; doi:10.3389/fimmu.2022.897879)
Supplement: Supplementary file 1 [file DataSheet_1.docx]

Supplementary Material

# Supplementary Figures and Tables

## Supplementary Figures


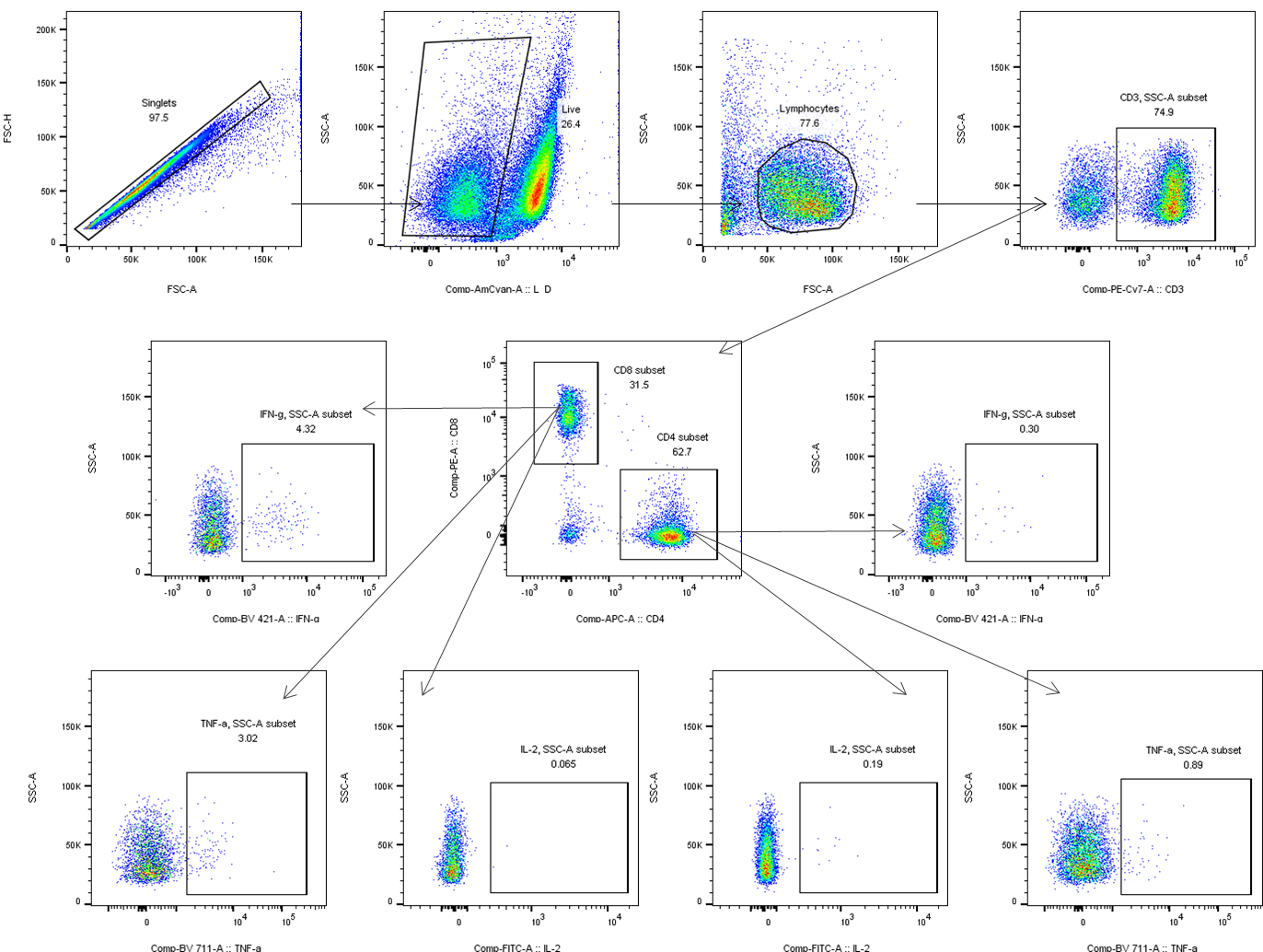


**Supplementary Figure 1** **Gating strategy of flow cytometry.** Briefly, single cells were identified from the total events via an FSC-H vs FSC-A gating. Next, a gating of FSC vs SSC was used to find the lymphocyte population. Then, CD3^+^ T cells were found via a gating of SSC vs CD3-PE-Cy7, which were further divided into CD3^+^CD4^+^ and CD3^+^CD8^+^ T cells via gating on CD4 vs CD8. Finally, frequencies of specific T cells were measured via gating on IL-2, TNF-a and IFN-γ, respectively.
