# Supplementary material for: Omicron Booster in Ancestral Strain Vaccinated Mice Augments Protective Immunities Against Both Delta and Omicron Variants
Source: Front Immunol. 2022 Jul 6;13:897879. doi: 10.3389/fimmu.2022.897879 (PMC9298979; doi:10.3389/fimmu.2022.897879)
Supplement: Supplementary file 2 [file Table_1.pdf]

Supplementary Table 1 The sequences of synthesized spike protein peptides used for T cell stimulation

Peptide	Sequence	Peptide	Sequence
Omicron_S_1	PFFSNVTWFHVISGTNGT	Wuhan_S_7	HSTQDLFLPFFSNVTWFH
Omicron_S_2	FHVISGTNGTKRFDNPVL	Wuhan_S_8	PFFSNVTWFHAIHVSGTN
Omicron_S_3	NPVLPFNDGVYFASIEKS	Wuhan_S_9	FHAIHVSGTNGTKRFDNP
Omicron_S_4	GVYFASIEKSNIIRGWIF	Wuhan_S_10	TNGTKRFDNPVLPFNDGV
Omicron_S_5	KVCEFQFCNDPFLDHKNN	Wuhan_S_11	NPVLPFNDGVYFASTEKS
Omicron_S_6	NDPFLDHKNNKSWMESE	Wuhan_S_12	GVYFASTEKSNIIRGWIF
Omicron_S_7	FKIYSKHTPIIVREPEDL	Wuhan_S_13	KSNIIRGWIFGTTLDSKT
Omicron_S_8	PIIVREPEDLPQGFSALE	Wuhan_S_14	IFGTTLDSKTQSLLIVNN
Omicron_S_9	FPNITNLCPFDEVFNATR	Wuhan_S_15	KTQSLLIVNNATNVVIKV
Omicron_S_11	CVADYSVLYNLAPFFTFK	Wuhan_S_16	NNATNVVIKVCEFQFCND
Omicron_S_12	YNLAPFFTFKCYGVSPTK	Wuhan_S_17	KVCEFQFCNDPFLGVYYH
Omicron_S_13	VIRGDEVRQIAPGQTGNI	Wuhan_S_18	NDPFLGVYYHKNNKSWME
Omicron_S_14	QIAPGQTGNIADYNYKLP	Wuhan_S_19	YHKNNKSWMESEFRVYSS
Omicron_S_15	LPDDFTGCVIAWNSNKLD	Wuhan_S_20	MESEFRVYSSANNCTFEY
Omicron_S_16	VIAWNSNKLDSKVSGNYN	Wuhan_S_21	SSANNCTFEYVSQPFLMD
Omicron_S_17	LDSKVSGNYNYLYRLFRK	Wuhan_S_22	EYVSQPFLMDLEGKQGNF
Omicron_S_19	YQAGNKPCNGVAGFNCYF	Wuhan_S_23	MDLEGKQGNFKNLREFVF
Omicron_S_20	NGVAGFNCYFPLRSYSFR	Wuhan_S_24	NFKNLREFVFKNIDGYFK
Omicron_S_21	YFPLRSYSFRPTYGVGHQ	Wuhan_S_25	VFKNIDGYFKIYSKHTPI
Omicron_S_22	FRPTYGVGHQPYRVVVLS	Wuhan_S_26	FKIYSKHTPINLVRDLPQ
Omicron_S_23	KCVNFNFNGLKGTGVLTE	Wuhan_S_27	PINLVRDLPQGFSALEPL
Omicron_S_24	GLKGTGVLTESNKKFLPF	Wuhan_S_28	PQGFSALEPLVDLPIGIN
Omicron_S_26	AVLYQGVNCTEVPVAIHA	Wuhan_S_29	PLVDLPIGINITRFQTLL
Omicron_S_28	CLIGAEYVNNSYECDIPI	Wuhan_S_30	INITRFQTLLALHRSYLT
Omicron_S_29	PIGAGICASYQTQTKSHR	Wuhan_S_31	LLALHRSYLTPGDSSSGW
Omicron_S_30	SYQTQTKSHRRARSVASQ	Wuhan_S_32	LTPGDSSSGWTAGAAAYY
Omicron_S_31	LLQYGSFCTQLKRALTGI	Wuhan_S_33	GWTAGAAAYYVGYLQPRT
Omicron_S_32	TQLKRALTGIAVEQDKNT	Wuhan_S_34	YYVGYLQPRTFLLKYNEN
Omicron_S_33	VKQIYKTPPIKYFGGFNF	Wuhan_S_35	RTFLLKYNENGTITDAVD
Omicron_S_34	PIKYFGGFNFSQILPDPS	Wuhan_S_36	ENGTITDAVDCALDPLSE
Omicron_S_35	LGDIAARDLICAQKFKGL	Wuhan_S_37	VDCALDPLSETKCTLKSF
Omicron_S_36	LICAQKFKGLTVLPPLLT	Wuhan_S_38	SETKCTLKSFTVEKGIYQ
Omicron_S_37	LGKLQDVVNHNAQALNTL	Wuhan_S_39	SFTVEKGIYQTSNFRVQP
Omicron_S_38	NHNAQALNTLVKQLSSKF	Wuhan_S_40	YQTSNFRVQPTESIVRFP
Omicron_S_39	TLVKQLSSKFGAISSVLN	Wuhan_S_41	QPTESIVRFPNITNLCPF
Omicron_S_40	KFGAISSVLNDIFSRLDK	Wuhan_S_42	FPNITNLCPFGEVFNATR
Wuhan_S_2	PLVSSQCVNLTTRTQLPP	Wuhan_S_43	PFGEVFNATRFASVYAWN
Wuhan_S_3	NLTTRTQLPPAYTNSFTR	Wuhan_S_44	TRFASVYAWNRKRISNCV
Wuhan_S_4	PPAYTNSFTRGVYYPDKV	Wuhan_S_45	WNRKRISNCVADYSVLYN
Wuhan_S_5	TRGVYYPDKVFRSSVLHS	Wuhan_S_46	CVADYSVLYNSASFSTFK
Wuhan_S_6	KVFRSSVLHSTQDLFLPF	Wuhan_S_47	YNSASFSTFKCYGVSPTK

Peptide	Sequence	Peptide	Sequence
Wuhan_S_48	FKCYGVSPTKLNDLCFTN	Wuhan_S_89	VAYSNNSIAIPTNFTISV
Wuhan_S_49	TKLNDLCFTNVYADSFVI	Wuhan_S_91	SVTTEILPVSMTKTSVDC
Wuhan_S_50	TNVYADSFVIRGDEVRQI	Wuhan_S_92	VSMTKTSVDCTMYICGDS
Wuhan_S_51	VIRGDEVRQIAPGQTGKI	Wuhan_S_93	DCTMYICGDSTECSNLLL
Wuhan_S_52	QIAPGQTGKIADYNYKLP	Wuhan_S_94	DSTECSNLLLQYGSFCTQ
Wuhan_S_53	KIADYNYKLPDDFTGCVI	Wuhan_S_95	LLQYGSFCTQLNRALTGI
Wuhan_S_54	LPDDFTGCVIAWNSNNLD	Wuhan_S_96	TQLNRALTGIAVEQDKNT
Wuhan_S_55	VIAWNSNNLDSKVGGNYN	Wuhan_S_97	GIAVEQDKNTQEVFAQVK
Wuhan_S_56	LDSKVGGNYNYLYRLFRK	Wuhan_S_98	NTQEVFAQVKQIYKTPPI
Wuhan_S_57	YNYLYRLFRKSNLKPFER	Wuhan_S_99	VKQIYKTPPIKDFGGFNF
Wuhan_S_58	RKSNLKPFERDISTEIYQ	Wuhan_S_100	PIKDFGGFNFSQILPDPS
Wuhan_S_59	ERDISTEIYQAGSTPCNG	Wuhan_S_101	NFSQILPDPSKPSKRSFI
Wuhan_S_60	YQAGSTPCNGVEGFNCYF	Wuhan_S_102	PSKPSKRSFIEDLLFNKV
Wuhan_S_61	NGVEGFNCYFPLQSYGFQ	Wuhan_S_103	FIEDLLFNKVTLADAGFI
Wuhan_S_62	YFPLQSYGFQPTNGVGYQ	Wuhan_S_104	KVTLADAGFIKQYGDCLG
Wuhan_S_63	FQPTNGVGYQPYRVVVLS	Wuhan_S_105	FIKQYGDCLGDIAARDLI
Wuhan_S_64	YQPYRVVVLSFELLHAPA	Wuhan_S_106	LGDIAARDLICAQKFNGL
Wuhan_S_65	LSFELLHAPATVCGPKKS	Wuhan_S_107	LICAQKFNGLTVLPPLLT
Wuhan_S_66	PATVCGPKKSTNLVKNKC	Wuhan_S_108	GLTVLPPLLTDEMIAQYT
Wuhan_S_67	KSTNLVKNKCVNFNFNGL	Wuhan_S_109	LTDEMIAQYTSALLAGTI
Wuhan_S_68	KCVNFNFNGLTGTGVLTE	Wuhan_S_110	YTSALLAGTITSGWTFGA
Wuhan_S_69	GLTGTGVLTESNKKFLPF	Wuhan_S_111	TITSGWTFGAGAALQIPF
Wuhan_S_70	TESNKKFLPFQQFGRDIA	Wuhan_S_112	GAGAALQIPFAMQMAYRF
Wuhan_S_71	PFQQFGRDIADTTDAVRD	Wuhan_S_113	PFAMQMAYRFNGIGVTQN
Wuhan_S_72	IADTTDAVRDPQTLEILD	Wuhan_S_114	RFNGIGVTQNVLYENQKL
Wuhan_S_73	RDPQTLEILDITPCSFGG	Wuhan_S_115	QNVLYENQKLIANQFNSA
Wuhan_S_74	LDITPCSFGGVSVITPGT	Wuhan_S_116	KLIANQFNSAIGKIQDSL
Wuhan_S_75	GGVSVITPGTNTSNQVAV	Wuhan_S_117	SAIGKIQDSLSSTASALG
Wuhan_S_76	GTNTSNQVAVLYQDVNCT	Wuhan_S_118	SLSSTASALGKLQDVVNQ
Wuhan_S_77	AVLYQDVNCTEVPVAIHA	Wuhan_S_119	LGKLQDVVNQNAQALNTL
Wuhan_S_78	CTEVPVAIHADQLTPTWR	Wuhan_S_120	NQNAQALNTLVKQLSSNF
Wuhan_S_79	HADQLTPTWRVYSTGSNV	Wuhan_S_121	TLVKQLSSNFGAISSVLN
Wuhan_S_80	WRVYSTGSNVFQTRAGCL	Wuhan_S_122	NFGAISSVLNDILSRLDK
Wuhan_S_81	NVFQTRAGCLIGAEHVNN	Wuhan_S_123	LNDILSRLDKVEAEVQID
Wuhan_S_82	CLIGAEHVNNSYECDIPI	Wuhan_S_124	DKVEAEVQIDRLITGRLQ
Wuhan_S_83	NNSYECDIPIGAGICASY	Wuhan_S_125	IDRLITGRLQSLQTYVTQ
Wuhan_S_84	PIGAGICASYQTQTNSPR	Wuhan_S_126	LQSLQTYVTQQLIRAAEI
Wuhan_S_85	SYQTQTNSPRRARSVASQ	Wuhan_S_127	TQQLIRAAEIRASANLAA
Wuhan_S_86	PRRARSVASQSIIAYTMS	Wuhan_S_128	EIRASANLAATKMSECVL
Wuhan_S_87	SQSIIAYTMSLGAENSVA	Wuhan_S_129	AATKMSECVLGQSKRVDF
Wuhan_S_88	MSLGAENSVAYSNNSIAI	Wuhan_S_130	VLGQSKRVDFCGKGYHLM

Peptide	Sequence	Peptide	Sequence
Wuhan_S_131	DFCGKGYHLMSFPQSAPH	Wuhan_S_144	LDSFKEELDKYFKNHTSP
Wuhan_S_132	LMSFPQSAPHGVVFLHVT	Wuhan_S_145	DKYFKNHTSPDVDLGDIS
Wuhan_S_133	PHGVVFLHVTYVPAQEKN	Wuhan_S_146	SPDVDLGDISGINASVVN
Wuhan_S_134	VTYVPAQEKNFTTAPAIC	Wuhan_S_147	ISGINASVVNIQKEIDRL
Wuhan_S_135	KNFTTAPAICHDGKAHFP	Wuhan_S_148	VNIQKEIDRLNEVAKNLN
Wuhan_S_136	ICHDGKAHFPREGVFVSN	Wuhan_S_149	RLNEVAKNLNESLIDLQE
Wuhan_S_137	FPREGVFVSNGTHWFVTQ	Wuhan_S_150	LNESLIDLQELGKYEQYI
Wuhan_S_138	SNGTHWFVTQRNFYEPQI	Wuhan_S_151	QELGKYEQYIKWPWYIWL
Wuhan_S_139	TQRNFYEPQIITTDNTFV	Wuhan_S_152	YIKWPWYIWLGFIAGLIA
Wuhan_S_140	QIITTDNTFVSGNCDVVI	Wuhan_S_156	CSCLKGCCSCGSCCKFDE
Wuhan_S_142	VIGIVNNTVYDPLQPELD	Wuhan_S_157	SCGSCCKFDEDDSEPVLK
Wuhan_S_143	VYDPLQPELDSFKEELDK	Wuhan_S_158	DEDDSEPVLKGVKLHYT

Note: Most neighboring peptides for Wuhan_S overlap by 10 aa except the following peptide pairs, Wuhan_S_89/91, Wuhan_S_140/142 and Wuhan_S_152/156, because few designed peptides could not be reliably synthesized. Omicron_S peptides were designed to cover the fragments containing mutations specific to the variant. The purity was beyond 95% for all peptides.
